# Bioethanol Steam Reforming for Hydrogen Production over Ni-Cr/SBA 15: Influence of Metal Loading and Ni/Cr Ratio

**DOI:** 10.3390/molecules30061206

**Published:** 2025-03-07

**Authors:** Pedro J. Megía, Lourdes García-Moreno, Arturo J. Vizcaíno, José A. Calles, Alicia Carrero

**Affiliations:** 1 Chemical and Environmental Engineering Group, Rey Juan Carlos University, Tulipán Street s/n, 28933 Móstoles, Madrid, Spain; pedro.megia@urjc.es (P.J.M.); lourdes.garcia@urjc.es (L.G.-M.); arturo.vizcaino@urjc.es (A.J.V.); alicia.carrero@urjc.es (A.C.); 2 Institute of Sustainable Technologies, Rey Juan Carlos University, Tulipán Street s/n, 28933 Móstoles, Madrid, Spain

**Keywords:** bioethanol, steam reforming, hydrogen, nickel, chromium, SBA-15, bimetallic catalyst

## Abstract

This work examines the influence of metal loading and the Ni/Cr ratio of Ni-Cr/SBA-15 catalysts on bioethanol steam reforming for the first time. The characterization of the synthesized samples reveals that higher Cr amounts result in lower Ni crystallite sizes due to the promoting effect of Cr, thereby enhancing the dispersion of the active phase. The catalytic performance has been evaluated in terms of ethanol conversion and H_2_ TOF (min^−1^). Ethanol conversion exhibits an increasing trend with higher Ni content, reaching up to 90% for samples containing 15 wt.%. By increasing the Cr content (lower Ni/Cr ratio) the results evidence a similar trend. A synergistic effect between Ni and Cr was appreciated in conversion values when the Ni content was below 11 wt.% and the Cr content exceeded 2 wt.%, which coincides with a smaller Ni crystallite size. Concerning the H_2_ TOF, the catalyst with the lowest Ni content (7 wt.%) exhibited a higher value with a notable enhancement upon increasing the Cr loading. However, a considerable decrease in H_2_ TOF was observed for samples with higher Ni loading. Therefore, the best catalytic performance, achieving nearly complete ethanol conversion and high hydrogen production, was reached when using catalysts with 7 wt.% Ni; the Cr loading should be increased to around 2 wt.%.

## 1. Introduction

Despite hydrogen being considered the energy vector of the future, the cleanness of its production is crucial to ensure this grade and to achieve the so-called Hydrogen Economy [1,2]. Nowadays, hydrogen is mostly produced using fossil fuels like natural gas steam reforming and coal gasification, contributing to global climate change. Moreover, despite fossil fuel prices having receded from their peaks in 2022, markets continue to exhibit tension and volatility [3]. Therefore, the development of new technologies based on renewable sources is a key role for its sustainability [4]. On this basis, numerous studies have focused their attention on an effective and environmentally friendly technology for its production [5].

Hydrogen production from biomass and/or residual wastes could be technically and economically feasible, given the current state of technology in developed countries [6]. In this regard, thermochemical processes are the most effective methods for producing hydrogen-rich gases from biomass [7,8]. More specifically, the steam reforming of biomass derivatives presents more benefits than pyrolysis and gasification from the point of view of production costs [9]. Compared to methane and other hydrocarbons, ethanol steam reforming (ESR) has the potential for widespread availability, low cost, and superior reactivity, which is the reason why it has been deeply studied in the literature [10]. Moreover, ethanol has a high hydrogen content, and, given that it is not toxic, its handling, storage, and transportation are safe [11]. These characteristics enable its distribution in a logistic net close to those used for conventional fuel stations.

ESR is an endothermic process, thus favored at high temperatures, which can be stoichiometrically represented as follows:

(1) $${\mathrm{CH}}_{3}{\mathrm{CH}}_{2}\mathrm{OH}+3H_{2}O\;↔\;2{\mathrm{CO}}_{2}+6H_{2}$$

The hydrogen yield is affected not only by the main reaction but also due to other side reactions that can lead to the formation of undesirable intermediated compounds or byproducts, whose prevalence is closely linked to the operating conditions and the design of the catalyst [12,13]. In this respect, Ni-based catalysts have been widely used in the literature because they have demonstrated promising results towards hydrogen selectivity and are cheaper than those based on noble metals. They have shown promising activity to facilitate the C-C and C-H bonds cleavage [14,15,16]. Unfortunately, Ni catalysts present some limitations, including deactivation by coke deposition and/or metal sintering, sensitivity to poisoning, and the lack of selectivity through the formation of undesirable byproducts. Perhaps the most significant drawback of Ni-based catalysts during steam reforming is their tendency to easily form coke deposits over the Ni crystallite surface. These carbon deposits may block active sites on the catalysts, decreasing their efficiency and ultimately causing deactivation, which also results in a decrease in hydrogen production [17,18]. These concerns have driven research into modifications of Ni catalysts, such as the incorporation of a second metal leading to bimetallic formulations to improve their catalytic stability and selectivity [19,20,21,22,23]. In previous works [22,23], we have reported the benefits of adding a second metal to Ni/SBA-15 in the steam reforming of various oxygenated hydrocarbons commonly found in aqueous streams resulting from thermochemical biomass treatments. More specifically, the combination of Ni and Cr, both in the catalyst’s formulation, exhibited higher conversion and hydrogen production when compared to Ni/SBA-15. Moreover, the coke deposited during the reforming reaction was drastically decreased, attributed to the formation of smaller Ni crystallites induced by the presence of Cr. Similarly, Bui et al. [24] evaluated plate Ni-Cr catalysts for the steam reforming of biogas, highlighting the beneficial role of Cr in preventing deactivation via sintering. This effect has also been reported elsewhere for other reforming reactions using Co-based catalysts [25,26]. Additionally, Cr has been employed as a promoter in different catalyst formulations to enhance the dispersion of the active phase, thereby preventing thermal sintering, while also creating oxygen vacancies due to its redox nature [27,28,29]. Rouibah et al. [30] prepared Ni-M (M = Cr, Mn, Co) catalysts for the dry reforming of methane. The presence of Cr in the catalyst formulation not only enhances the catalytic activity but also stabilizes the active Ni phase and potentially reduces coke formation, thus achieving the best catalytic performance. Ramantani et al. [31] studied the incorporation of different transition metals to a Ni-based perovskite, concluding that the presence of Cr not only improved the structural stability but also increased the oxygen vacancies and reduced coke formation in the steam reforming of propane. For their part, Abrokwah et al. [32] studied the effect of Cr and CeO_2_ addition to Ni/SBA-15 in the steam reforming of glycerol. They concluded that chromium has a dual effect in the glycerol steam reforming since it negatively impacts glycerol conversion and hydrogen selectivity in the short-term time-on-stream but, in the long-term, it significantly enhances the stability of the catalyst by preventing sintering and structural collapse. To our knowledge, there are no studies that have evaluated the Cr content in the catalytic behavior of bimetallic Ni-based catalysts. Furthermore, over the past two decades, the Scopus database shows that less than 15 articles have been published on bimetallic Ni-Cr catalysts for steam reforming, with none of them specifically addressing bioethanol as a feedstock. Therefore, the aim of the present work is the preparation of different Ni-Cr/SBA-15 catalysts with varying metal content and Ni/Cr ratios to optimize their catalytic properties towards hydrogen production in the ESR reaction.

## 2. Results and Discussion

### 2.1. Catalysts Characterization

**Figure 1:**
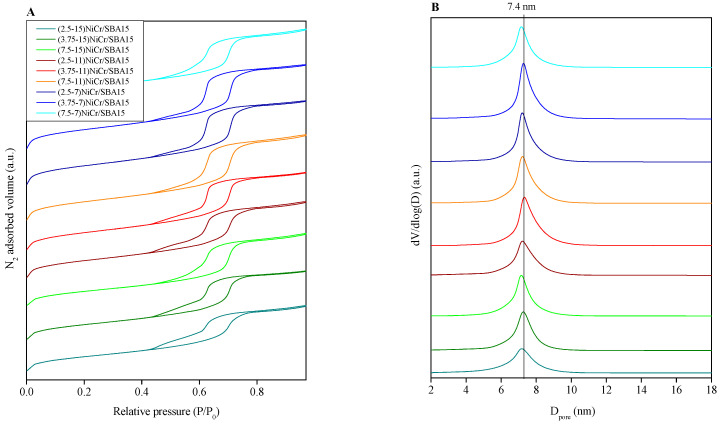
(**A**) N_2_ adsorption–desorption isotherms and (**B**) pore size distribution of the calcined Ni-Cr catalysts.

N_2_ adsorption–desorption isotherms, displayed in Figure 1A for all the prepared Ni-Cr/SBA-15 catalysts, show a type IV isotherm with an H1-hysteresis loop in all cases. According to the IUPAC, this isotherm model is characteristic of mesoporous materials with hexagonal pore arrangements. This indicates the preservation of the initial SBA-15 structure after the metal incorporation. However, as the metal content increases, some distortion of the porous structure can be assumed from the shape of the isotherm, and less nitrogen is adsorbed, which suggests some loss of porosity given the higher metal content. The pore size distribution obtained using the BJH method is shown in Figure 1B. It seems clear that the pore size distribution curve is narrower when the metal content is the smallest. The peaks are widened as a result of the partial blockage of the mesopores with increasing metal content, thus corroborating some distortion of the porous structure. The textural properties determined from the N_2_-physisorption isotherms of calcined catalysts are summarized in Table 1 along with other physicochemical properties. As the metal content increases, there is a notable decrease in both the BET surface area and pore volume, even though these values were high for all the samples.

**Figure 2:**
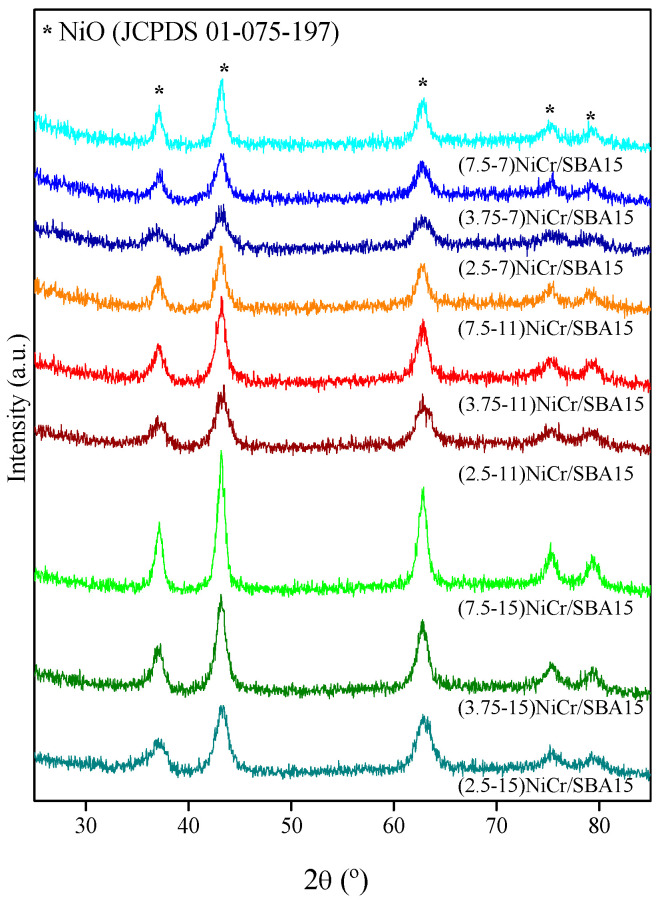
XRD patterns of calcined Ni-Cr-based SBA-15 catalysts.

Diffraction patterns of calcined Ni-Cr/SBA-15 catalysts are depicted in Figure 2. Peaks at 2θ = 37.2, 43.3, 63.0, 75.5, and 79.2° corresponding to (111), (200), (220), (311), and (222) planes of the cubic phase of NiO (JCPDS 01-075-0197), respectively, arise in all the samples. No peaks ascribed to any Cr species are possible to be discerned in the calcined catalysts. This observation aligns with results reported by other authors when incorporating Cr as a promoter [35]. This effect could be related to the higher Ni content compared to the Cr content, which can contribute to the lower intensity of Cr-oxides peaks, especially at low loadings, as previously reported [22,26], or because the formation of NiCr_2_O_4_ (JCPDS 01-088-0109) spinel, which could not be detected by XRD due to the overlap with the main lines of the NiO pattern. It is noticeable that with an increase in the Ni content in the catalyst, formulation peaks become narrower, highlighting the formation of larger crystallites size. This was verified with the results calculated using the Scherrer equation in the (200) plane of cubic NiO (see Table 1). On the contrary, when the Cr content increases, peaks corresponding to NiO become wider, leading to smaller crystallite sizes. This is in line with that reported in the literature since Cr has been used as a promoter agent to increase dispersion in metal-supported catalysts [36].

**Figure 3:**
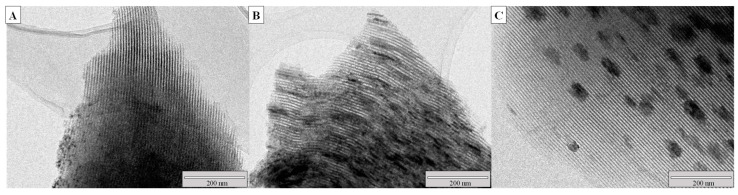
TEM micrographs of calcined (**A**) (2.5-15)NiCr/SBA15, (**B**) (3.75-15)NiCr/SBA15, and (**C**) (7.5-15)NiCr/SBA15 catalysts.

TEM images of samples with a 15 wt.% of Ni, varying the Ni/Cr ratio between 2.5 and 7.5, are displayed in Figure 3 to give an overview of the catalyst morphology and active phase dispersion over the SBA-15 support. The micrographs evidence the well-ordered hexagonal array of cylindrical channels characteristic of SBA-15 material, agreeing with the results obtained by N_2_ physisorption isotherms. Moreover, dark zones can be observed on the mesoporous structure of SBA-15, corresponding to Ni and Cr oxides. As the Ni/Cr ratio decreases, the oxide particles diminish, thereby confirming a higher dispersion of NiO particles in agreement with the mean crystallite sizes calculated from the XRD patterns (see Table 1).

**Figure 4:**
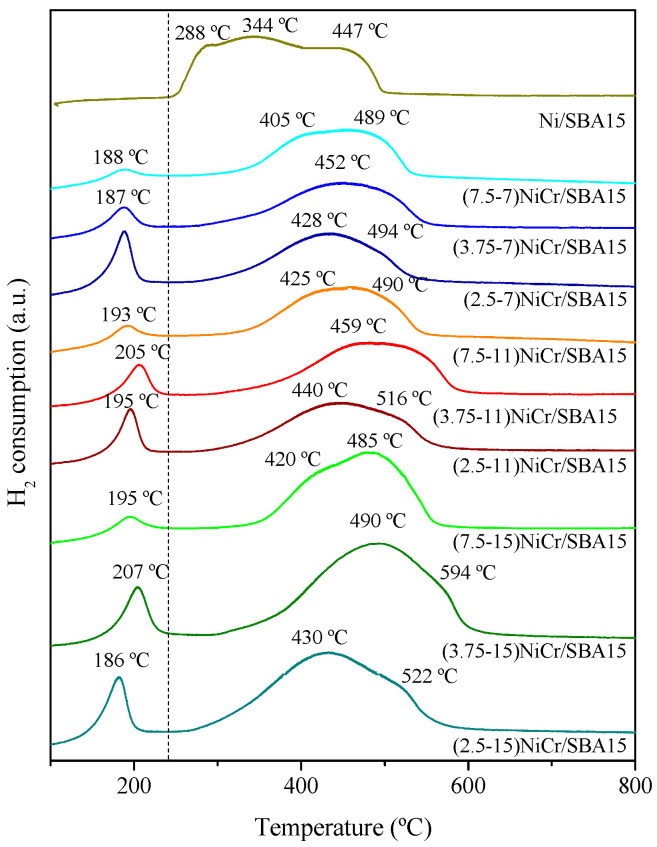
H_2_-TPR profiles of calcined SBA-15-supported bimetallic Ni-Cr catalysts compared to Ni/SBA-15 reduction profile from refs. [23,37].

To study the reduction behavior of the oxide species in the calcined catalysts, H_2_-TPR analyses were performed, as shown in Figure 4. For comparative purposes, the reduction profile of Ni from refs [22,23] has been included in which a single reduction zone with three different features appears at 288, 344, and 447 °C related to NiO particles with varying degrees of interaction with the support. Conversely, in the Ni-Cr samples, two different regions can be distinguished. The first one at lower temperatures (180–210 °C) is related to the reduction in Cr-species to Cr^3+^ affected by the presence of NiO [37,38,39]. As it can be observed, higher Cr content resulted in an increase in the reduction area for this peak since more Cr species are being reduced. On the other hand, the second reduction zone at higher temperatures (with maxima > 400 °C) is ascribed to the reduction in NiO species with different degrees of interaction with the support to Ni^0^, as evidenced by different maxima in the reduction profile for each sample [40,41]. A shoulder can be distinguished at temperatures close to 500 °C, which could be related to the spinel NiCr_2_O_4_, as reported elsewhere [42,43], where Cr_2_O_3_ would be forming part of the mixed oxide having strong interaction with NiO. This feature is more intense when referred to samples with the highest Ni/Cr ratio (7.5) along with the lowest area for the reduction region at low temperatures (180–210 °C). This may indicate that, for these catalysts, Cr is preferably taking part in the spinel.

Finally, as expected, samples with higher Ni metal content resulted in increased H_2_ consumption, as summarized in Table 2, since more Ni oxide species are available. However, in all the cases, the reducibility ranged between 94 and 99%, indicating that all the samples achieved a similar level of NiO reduction.

**Figure 5:**
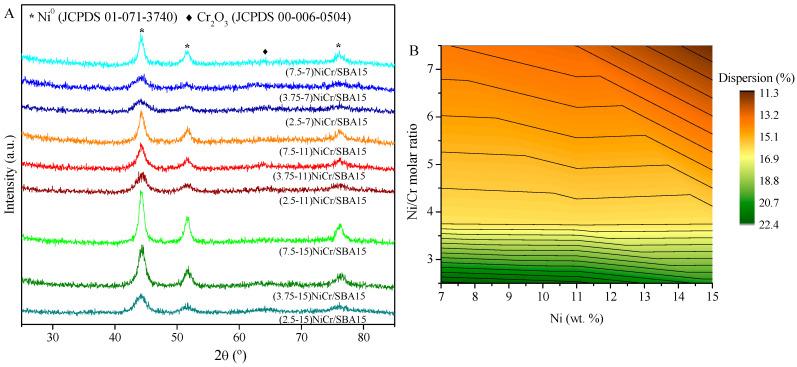
(**A**) XRD of reduced Ni-Cr catalysts at 700 °C and (**B**) Ni dispersion as a function of Ni and Cr content.

Once reduced at 600 °C, all the catalysts were characterized again by XRD measurements. As it can be seen from Figure 5A, no diffraction lines of Ni- or Cr-oxides species can be differentiated. On the one hand, regarding Ni-oxides, the absence of NiO diffraction lines indicates complete reduction during the activation process. Instead, peaks corresponding to the (111), (200), and (220) planes of the cubic phase of the Ni^0^ pattern (JCPDS 01-071-3740) can be appreciated, showing the reflection at 2θ = 44.5, 51.8, and 74.4°, respectively. On the other hand, the absence of peaks ascribed to Cr^0^ can be explained by the reducibility of Cr_2_O_3_, which is hampered due to its extraordinarily high and negative energy of formation [44]. Accounting for the presence of Cr_2_O_3_ peaks, a change in the baseline could be discerned, which could be attributed to the presence of the rhombohedral Cr_2_O_3_ crystal system (JCPDS 00-006-0504) at 2θ = 63.4°. Hence, the high dispersion of these species over the SBA-15 support is reaffirmed. Ni^0^ crystallite sizes were estimated using the Scherrer equation from the diffraction plane (111). The dispersion of the Ni crystallites is displayed in Figure 5B as a function of Ni/Cr ratio and Ni content.

To summarize, as the metallic (Ni+Cr) content increases, the specific surface area of the catalyst decreases. Regarding the Ni^0^ crystallite size, when the Ni content increases, the crystallite size also increases. By contrast, when the Ni/Cr ratio decreases (higher Cr content), the mean crystallite size decreases. Following these trends, ethanol steam reforming should be favored using catalysts with lower metallic content and lower Ni/Cr ratios to achieve smaller Ni particles, leading to more accessible active sites.

### 2.2. Ethanol Steam Reforming Tests

**Figure 6:**
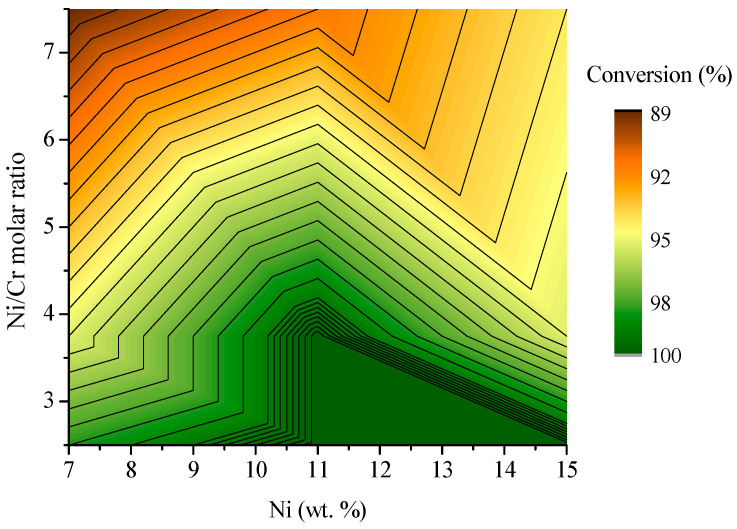
Ethanol conversion after 5 h time-on-stream as a function of Ni and Cr content (S/C = 1.85, T = 600 °C, P = atm, WHSV = 16.6 h^−1^).

The catalytic performance of the prepared catalysts was evaluated in the bioethanol steam reforming reaction through ethanol conversion and TOF, as described in the experimental section. Figure 6 shows the 3D surface distribution for ethanol conversion after 5 h time-on-stream as a function of the Ni and Cr content in wt.%. All the samples reached conversions above 85%. Considering only the Ni content, as it increases, the ethanol conversion also rises, reaching a value close to 90% with a 15 wt.% of Ni. Similarly, the ethanol conversion increases with the Cr content, ascribed to higher Ni dispersion. It is possible to observe a region in which the ethanol conversion is almost complete when the Ni content is below 11 wt.% and the Cr content is above 2 wt.%. This area perfectly aligns with the zone with smaller Ni crystallite sizes represented in Figure 5B. According to what is reported in the literature, lower crystallite sizes may lead to better catalytic performance [45]. In line with this, Gupta et al. [46] studied the effect of the metal amount over Ni/Al_2_O_3_ catalysts in the tri-reforming of methane, concluding that catalytic performance was closely related to metal dispersion. Considering that Cr is significantly cheaper compared to nickel, to reach 100% ethanol conversion, it would be more economically viable to reduce the nickel percentage to 7 wt.% along with a 2 wt.% of Cr. Furthermore, this combination represents the sample with the highest dispersion of Ni (see Table 1). In this respect, in previous works [23], it was verified that the effect of Cr promotion on the Ni-crystallite sizes avoided the formation of intermediary surface species of coke deposition reaching the best catalytic performance in the steam reforming of model bio-oil aqueous fraction. In another study [26], it was verified that adding Cr to Co/SBA-15 resulted in lower Co crystallites, thus improving the metal dispersion and increasing the activity in acetic acid steam reforming. Similarly, Singha et al. [47] studied the effect of Pd promotion on Ni/MgO for methane dry reforming. They concluded that the increased catalytic activity achieved by promoting Ni catalysts with a small amount of Pd was related to an increase in Ni reducibility and dispersion.

**Figure 7:**
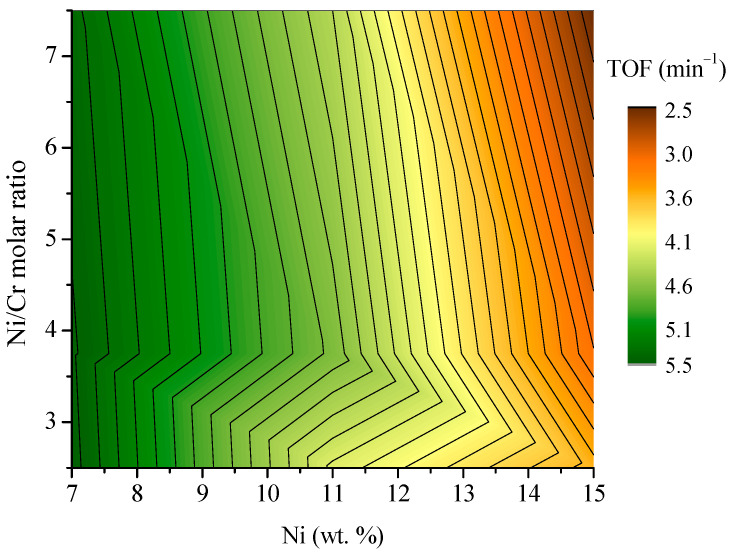
H_2_ TOF (min^−1^) after 5 h time-on-stream as a function of Ni and Cr content (S/C = 1.85, T = 600 °C, P = atm, WHSV = 16.6 h^−1^).

The effect of the Ni and Cr content in the catalyst formulation was also evaluated through the H_2_ TOF (min^−1^), defined as the ratio between the H_2_ formation rate and the number of Ni moles in the catalysts. The obtained 3D surface distribution as a function of Ni content in wt.% and the Ni/Cr molar ratio is displayed in Figure 7. For high Ni contents, close to 15 wt.%, the H_2_ TOF decreases considerably, resulting in values below 3.5 min^−1^ in all cases. This effect is related to the hydrogen production, which was not significantly improved for these samples, while the amount of Ni in the catalyst formulation was doubled compared to catalysts with a 7 wt.% of Ni. Conversely, as the Ni content decreases in the catalyst, the H_2_ TOF value increases, reaching the maximum values of 5.5–6.0 min^−1^ for the catalysts with a 7 wt.% of Ni. Concerning the Ni/Cr ratio for these samples, an increase in the TOF value can be observed at lower values ascribed to the higher ethanol conversions obtained (see Figure 6).

Apart from hydrogen, other byproducts are formed during bioethanol steam reforming using Ni-Cr catalysts. In this regard, Table 3 shows, along with hydrogen selectivities, the byproducts distribution, including CO_2_, CO, CH_4_, and coke. Regarding the distribution of gaseous products, no significant differences can be highlighted among the catalysts tested in the bioethanol reforming reaction. In all cases, the molar percentages were similar, with CO_2_ ranging approximately between 20% and 22%, CO between 13% and 15%, and CH_4_ between 3% and 6%. These small variations can be attributed to the different metal amounts in the catalysts, which influence the extent to which certain reactions are favored within the complex network of reactions involved in bioethanol reforming. Concerning coke deposition, in all cases, the amount was very low since the presence of Cr provides high resistance to coke formation because it inhibits the Ni active sites encapsulation by the carbon formation [48]. A significant reduction in coke deposition was observed in all cases with the lowest Ni/Cr ratio, highlighting the positive impact of including Cr in the catalyst formulation. This reduction in coke formation is in line with the smaller maximum temperature obtained in the DTG profiles summarized in Table 3. In all the cases, the maximum temperature is between 510 and 540 °C, suggesting the formation of carbon nanofilaments with different ordering degrees since amorphous carbon oxidizes at lower temperatures [49]. The lower the temperature, the more disordered the coke structures become, making its gasification easier in the reaction environment. This assumption aligns with the coke deposited after 5h time-on-stream, since for a Ni/Cr ratio of 2.5—regardless of the Ni content—the lower temperature is achieved, which explains the smaller amount of coke deposited over these samples. Furthermore, the mean crystallite sizes of the spent catalysts estimated by applying the Scherrer equation on the main diffraction line on Ni^0^ in the spent catalysts (Figure S1) are also displayed in Table 3. For all the catalysts tested, the increase in the Ni^0^ size is below 10% compared to the reduced samples, thus stating that the sintering effect under the operating conditions has a minor effect.

Based on the results described above, to select the sample with the best catalytic performance based on the metal content and the Ni/Cr ratio, attention should be paid to the hydrogen production and the ethanol conversion. In this regard, considering the results shown in Figure 6 and Figure 7, it can be established that 7 wt.% of Ni will increase the H_2_ TOF value. To maximize the conversion, the amount of Cr in the catalyst formulation should be increased up to 2 wt.% (Ni/Cr = 3.75) to achieve conversions close to 100%, thus optimizing the catalytic performance of the Ni-Cr/SBA-15 catalysts in the steam reforming of ethanol.

## 3. Materials and Methods

Catalysts containing different concentrations of Ni and Cr were prepared by incipient wetness impregnation over SBA-15 material used as support using mixed aqueous solutions of Ni(NO_3_)_2_·6H_2_O (Scharlau, Barcelona, Spain) and Cr(NO_3_)_3_·9H_2_O (Aldrich, St. Louis, MO, USA) as metal precursors. The prepared catalysts were subsequently calcined under airflow up to 600 °C with a heating ramp of 2 °C/min. Samples were named (X-Y)NiCr/SBA15, where X refers to the molar Ni/Cr ratio (2.5–7.5) while Y refers to the amount of Ni in terms of wt.% (7–15). Calcined catalysts were characterized using different techniques, such as XRD, N_2_-physisorption, ICP-AES, H_2_-TPR, and TEM, to evaluate their textural and chemical properties.

X-ray diffraction (XRD) measurements were recorded using an X’pert PRO diffractometer (Philips, Eindhoven, The Netherlands) with CuKα radiation. The Scherrer equation was used to estimate the mean crystallite size based on the obtained diffractograms. N_2_ adsorption–desorption isotherms were acquired on a Micromeritics Tristar 3000 analyzer (Micromeritics, Norcross, GA, USA) at 77 K, from which specific surface areas were estimated using the Brunauer–Emmett–Teller model (BET). Pore volumes were estimated by applying the Barret–Joyner–Halenda model (BJH) from the desorption branch. Before the analysis, all the samples were outgassed under vacuum for 4 h at 200 °C. Inductively coupled plasma atomic emission spectroscopy (ICP-AES) was used to determine the Ni and Cr contents, using a Varian VISTA-PROAX spectrophotometer (Varian, Palo Alto, CA, USA). The temperature-programmed reduction (H_2_-TPR) technique was used to evaluate the reducibility of the prepared catalysts. The reduction profiles were recorded on a Micromeritics AUTOCHEM 2910 apparatus (Micromeritics, Norcross, GA, USA), reducing the samples in 10% H_2_/Ar with a heating rate of 5 °C/min until 980 °C. The transmission electron microscopy (TEM) technique gives information about the catalyst morphology. TEM micrographs were acquired on a Philips TECNAI 20 microscope (200 kV; Philips, Eindhoven, The Netherlands) with a resolution of 0.28 nm after sample preparation, which involved its suspension in acetone and subsequent deposition on a carbon-coated copper grid.

ESR reactions were performed in a Microactivity PRO unit (PID Eng. & Tech; Alcobendas, Madrid, Spain) equipped with a stainless steel 316 (i.d. = 9.2 mm, L = 300 mm) fixed-bed reactor located in an electric oven. The reactions were carried out isothermally at 600 °C (measured employing a K-type thermocouple) under atmospheric pressure. The samples were previously reduced with pure hydrogen flow according to the H_2_-TPR results. The ethanol mixture (steam-to-carbon molar ratio of 1.85) was pumped at WHSV = 16.6 h^−1^ and then mixed with N_2_ (60 mL/min), used as a diluent and internal standard. The gas reaction products were analyzed online using an Agilent 490 Micro-GC (Agilent, Santa Clara, CA, USA) equipped with a thermal conductivity detector (TCD), a PoraPlot U column (10 m), and a Molecular Sieve 5A column (20 m) using He and Ar as carrier gas, respectively. Condensable vapors were collected in a condensing unit based on the Peltier effect and subsequently analyzed in a Varian CP-3900 chromatograph equipped with a CP-WAX 52 CB column (30 m × 0.25 mm, DF = 0.25) and flame ionization detector (FID) using 1,4-butanediol as internal standard. The catalytic performance was evaluated in terms of ethanol conversion (X_EtOH_, Equation (2)) and H_2_ turnover frequency (TOF, Equation (3)), where F_i_ is related to the molar flowrate in the inlet/outlet, and mol_Ni_ is the total amount of Ni (mol) in the catalyst formulation.

(2) $$X_{\mathrm{EtOH}}(\%)=\frac{F_{\mathrm{EtOH},\mathrm{in}}-F_{\mathrm{EtOH},\mathrm{out}}}{F_{\mathrm{EtOH},\mathrm{in}}}\cdot 100$$

(3) $$\mathrm{TOF}\;(\min^{-1})=\frac{F_{H_{2},\mathrm{out}}}{{\mathrm{mol}}_{\mathrm{Ni}}}$$

## 4. Conclusions

The effect of the metal content and the Ni/Cr ratio over Ni-Cr/SBA-15 catalysts has been evaluated for the first time in the steam reforming of bioethanol. Increasing the Cr content enhances the Ni dispersion, as evidenced by the reduction in mean crystallite sizes calculated from the main X-ray diffraction lines of NiO and Ni^0^ in the calcined and reduced samples, respectively. As a result, increasing the Cr loading resulted in higher bioethanol conversion. This behavior is a consequence of the higher amount of active sites due to the promotion of Ni dispersion by Cr. On the other hand, the hydrogen production, measured in terms of TOF (min^−1^), decreases with the Ni content, suggesting that increased metal content does not necessarily improve hydrogen production. In fact, for the samples with the highest Ni content, TOF values remained below 3.5 min^−1^ in all cases. Nevertheless, for these samples, increasing the Cr content led to improved hydrogen production due to higher ethanol conversion. These findings suggest that lower Ni contents (7 wt.%) can increase the H_2_ TOF but, in order to maximize the bioethanol conversion and the hydrogen production, the amount of Cr in the catalyst formulation should be increased to around 2 wt.%. The optimization of the metal content and the Ni/Cr ratio can potentially lead to near-complete ethanol conversion with high hydrogen production, thereby optimizing the catalytic performance of Ni-Cr/SBA-15 catalysts in the steam reforming of bioethanol.

## Figures and Tables

**Table 1:** Physicochemical properties of Ni-Cr/SBA-15 catalysts.

Catalyst	Ni (wt.%) ^a^	Cr (wt.%) ^a^	S_BET_ (m^2^/g)	Vpore (cm^3^/g) ^b^	Dpore (nm) ^c^	d NiO (nm) ^d^	d Ni (nm) ^e^	Ni Dispersion (%) ^f^
(7.5-7)NiCr/SBA15	6.4	1.0	534	0.82	7.1	7.8	7.3	13.8
(3.75-7)NiCr/SBA15	7.0	1.8	539	0.80	7.3	7.6	6.2	16.3
(2.5-7)NiCr/SBA15	7.1	2.7	527	0.79	7.2	5.0	4.5	22.4
(7.5-11)NiCr/SBA15	10.8	1.1	527	0.81	7.2	7.9	7.6	13.3
(3.75-11)NiCr/SBA15	11.0	2.7	513	0.74	7.3	7.3	6.8	16.2
(2.5-11)NiCr/SBA15	10.6	4.1	496	0.73	7.2	5.3	4.6	22.0
(7.5-15)NiCr/SBA15	14.4	1.8	461	0.70	7.2	9.5	8.9	11.3
(3.75-15)NiCr/SBA15	14.8	3.8	463	0.67	7.3	7.0	6.2	16.3
(2.5-15)NiCr/SBA15	14.7	5.7	444	0.64	7.2	5.7	4.9	20.6

^a^ Determined by ICP-AES in calcined samples; ^b^ measured at P/P_0_ = 0.97; ^c^ maximum of the BJH pore size distribution; ^d^ determined from XRD of calcined catalysts by Scherrer equation from the (200) diffraction plane of NiO; ^e^ determined from XRD of reduced catalysts by Scherrer equation from the (111) diffraction plane of Ni^0^; ^f^ dispersion calculated using the equation D (%) = 101/DNi assuming spherical nickel particles [33,34].

**Table 2:** Summary of theoretical consumption for NiO reduction and experimental H_2_-TPR uptake over Ni-Cr catalysts.

Catalyst	H_2_ Consumption (μmol H_2_)	
	Theoretical	Experimental
(7.5-7)NiCr/SBA15	109.0	102.3
(3.75-7)NiCr/SBA15	105.6	99.3
(2.5-7)NiCr/SBA15	121.0	114.3
(7.5-11)NiCr/SBA15	184.0	175.8
(3.75-11)NiCr/SBA15	187.4	178.2
(2.5-11)NiCr/SBA15	180.6	171.2
(7.5-15)NiCr/SBA15	245.3	236.5
(3.75-15)NiCr/SBA15	252.2	249.3
(2.5-15)NiCr/SBA15	250.5	246.4

**Table 3:** H_2_ selectivity, byproduct distribution in the ethanol steam reforming using Ni-Cr catalysts after 5 h time-on-stream (S/C = 1.85, T = 600 °C, P = atm, WHSV = 16.6 h^−1^), and spent catalysts characterization.

Catalyst	S H_2_ (%)	Product Distribution (mol %)			Coke (g_coke_/g_cat_·h)	T_DTG, max_ (°C)	d Ni ^a^ (nm)
		CO_2_	CO	CH_4_			
(7.5-7)NiCr/SBA15	52.2	20.4	13.8	4.0	0.008	531	7.6
(3.75-7)NiCr/SBA15	55.3	20.3	14.3	5.3	0.008	526	6.7
(2.5-7)NiCr/SBA15	57.8	20.8	15.6	3.4	0.006	512	4.8
(7.5-11)NiCr/SBA15	61.1	21.3	13.1	2.9	0.012	538	8.3
(3.75-11)NiCr/SBA15	63.1	21.2	12.3	6.1	0.013	532	7.1
(2.5-11)NiCr/SBA15	58.9	20.6	15.1	5.5	0.003	518	4.9
(7.5-15)NiCr/SBA15	59.0	21.1	14.9	3.2	0.018	540	9.7
(3.75-15)NiCr/SBA15	62.9	21.8	12.7	5.4	0.014	519	6.2
(2.5-15)NiCr/SBA15	63.8	21.9	14.1	2.6	0.003	511	5.1

^a^ Determined from XRD of used catalysts by Scherrer equation from the (111) diffraction plane of Ni^0^.

## Data Availability

The original contributions presented in this study are included in the article. Further inquiries can be directed to the corresponding author.

## Supplementary Materials

The following supporting information can be downloaded at: https://www.mdpi.com/article/10.3390/molecules30061206/s1, Figure S1: XRD of spent Ni-Cr Catalysts after 5h time-on-stream (S/C=1.85, T = 600 °C, P = atm, WHSV = 16.6h^−1^).
