# Supplementary material for: Bioethanol Steam Reforming for Hydrogen Production over Ni-Cr/SBA 15: Influence of Metal Loading and Ni/Cr Ratio
Source: Molecules. 2025 Mar 7;30(6):1206. doi: 10.3390/molecules30061206 (PMC11945284; doi:10.3390/molecules30061206)
Supplement: Supplementary file 1 [file molecules-30-01206-s001.zip › molecules-3489599-supplementary.pdf]

Supplementary Material:

Bioethanol steam reforming for hydrogen production over Ni-Cr/SBA 15: Influence of metal loading and Ni/Cr ratio

P.J. Megía [1], L. García-Moreno [1], A.J. Vizcaíno [1], J.A. Calles [1,2,*], A. Carrero [1,2]

[1] Chemical and Environmental Engineering Group. ESCET, Rey Juan Carlos University. Tulipan street s/n. 28933 Mostoles, Madrid, Spain.

[2] Institute of Sustainable Technologies. Rey Juan Carlos University, Tulipan street s/n. 28933 Mostoles, Madrid, Spain.

*Corresponding author: joseantonio.calles@urjc.es (J.A. Calles)

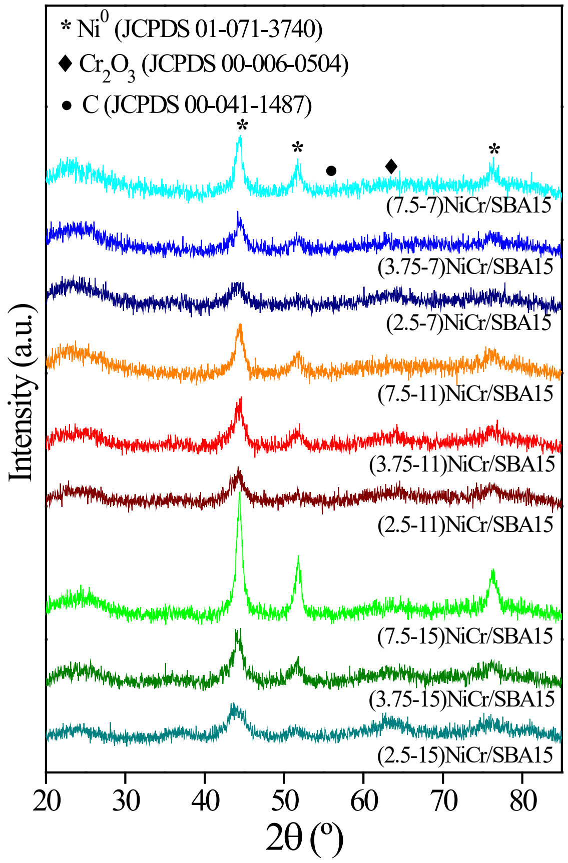

Figure S1. XRD of spent Ni-Cr Catalysts after 5h time-on-stream (S/C=1.85, T=600°C, P= atm, WHSV=16.6h^{-1}).
